# Development of an aversive Pavlovian-to-instrumental transfer task in rat

**DOI:** 10.3389/fnbeh.2013.00176

**Published:** 2013-11-26

**Authors:** Vincent Campese, Margaret McCue, Gabriel Lázaro-Muñoz, Joseph E. LeDoux, Christopher K. Cain

**Affiliations:** ^1^Center for Neural Science, New York UniversityNew York, NY, USA; ^2^Emotional Brain Institute, Nathan Kline Institute for Psychiatric ResearchOrangeburg, NY, USA

**Keywords:** Pavlovian, instrumental, transfer, avoidance, shuttling, rat

## Abstract

Pavlovian-to-instrumental transfer (PIT) is an effect whereby a classically conditioned stimulus (CS) enhances ongoing instrumental responding. PIT has been extensively studied with appetitive conditioning but barely at all with aversive conditioning. Although it's been argued that conditioned suppression is a form of aversive PIT, this effect is fundamentally different from appetitive PIT because the CS suppresses, instead of facilitates, responding. Five experiments investigated the importance of a variety of factors on aversive PIT in a rodent Sidman avoidance paradigm in which ongoing shuttling behavior (unsignaled active avoidance or USAA) was facilitated by an aversive CS. Experiment 1 demonstrated a basic PIT effect. Experiment 2 found that a moderate amount of USAA extinction produces the strongest PIT with shuttling rates best at around 2 responses per minute prior to the CS. Experiment 3 tested a protocol in which the USAA behavior was required to reach the 2-response per minute mark in order to trigger the CS presentation and found that this produced robust and reliable PIT. Experiment 4 found that the Pavlovian conditioning US intensity was not a major determinant of PIT strength. Experiment 5 demonstrated that if the CS and US were not explicitly paired during Pavlovian conditioning, PIT did not occur, showing that CS-US learning is required. Together, these studies demonstrate a robust, reliable and stable aversive PIT effect that is amenable to analysis of neural circuitry.

## Introduction

Much has been learned about the neural basis of aversive conditioning through studies of Pavlovian threat (fear) conditioning (PTC) (e.g., Maren, 2005; Johansen et al., 2011a,b). This work shows how learned threats come to elicit innate reactions, such as freezing behavior and supporting physiological responses. But a conditioned stimulus (CS) such as this not only elicits reactions, it also motivates actions that allow the organism to use instrumental behaviors to help cope with the threating situation. Most often the relation between aversive Pavlovian and instrumental learning is studied using avoidance conditioning (e.g., Sarter and Markowitsch, 1985; Gabriel et al., 2003; Choi et al., 2010). But in such tasks the Pavlovian and instrumental training are intermixed and it becomes procedurally difficult to separate the effects of the Pavlovian CS on the instrumental response. A more effective way to study the motivational influences of a Pavlovian CS on instrumental behavior is to use Pavlovian-to-instrumental transfer (PIT) tasks in which the Pavlovian CS modulates a separately trained instrumental response (Estes, 1948; Lovibond, 1983; Rescorla, 1994; Corbit and Balleine, 2005, 2011). Because Pavlovian and instrumental training occur separately, PIT provides an experimental procedure in which the effects of conditioned motivation can be evaluated in isolation from primary reinforcement processes. Maintaining these psychological processes as distinct is important for understanding how they are mediated by the brain.

In contrast to PTC, little attention has been paid to aversive PIT. However, appetitive PIT has been studied extensively (see Holland and Gallagher, 2003; Corbit and Balleine, 2005, 2011; Holmes et al., 2010; Shiflett and Balleine, 2010; Wiltgen et al., 2012). For example, rats exhibit an increase in instrumental responding (e.g., lever press) for food when presented with a CS (e.g., tone) that predicts food. This effect can be understood either in terms of sensory-specific processes or general motivational processes, mostly depending on whether the unconditioned stimuli (USs) used in the Pavlovian and instrumental training phases match or not-but also on the extent of instrumental choice during a PIT test (see Holmes et al., 2010; but also see Hall et al., 2001; Holland and Gallagher, 2003). Further, much information about the neural circuits that contribute to appetitive PIT has been acquired (Cardinal et al., 2002; Holland and Gallagher, 2003; Balleine and Killcross, 2006, etc). These findings are sometimes assumed to be applicable to aversive PIT as well (Balleine and Killcross, 2006). Evidence for supporting the similarity of appetitive and aversive PIT mainly comes from studies of the conditioned suppression task (Estes and Skinner, 1941; Hunt and Brady, 1951; Killcross et al., 1997). While conditioned suppression is in some sense a form of aversive transfer this effect is fundamentally different from appetitive PIT because in suppression (a) the US is different in the Pavlovian and instrumental phases and (b) the CS suppresses, instead of facilitates responding. To directly compare the brain systems involved in appetitive and aversive PIT, the PIT procedure should involve an aversively motivated instrumental behavior.

In order to better understand aversive Pavlovian-instrumental interactions in behavior, and to draw comparisons across appetitive and aversive tasks in terms of brain mechanisms, it would be best to use a version of PIT that is more comparable to appetitive PIT. This would involve a task in which the US has the same motivational significance in the Pavlovian and instrumental training phases. Previous behavioral studies suggest this is possible (Bolles and Popp, 1964; Rescorla and LoLordo, 1965; Rescorla, 1968; Weisman and Litner, 1969; Overmier and Payne, 1971; Overmier and Brackbill, 1977; Patterson and Overmier, 1981). These studies found that a Pavlovian CS conditioned with shock enhanced an aversive instrumental response—unsignaled Sidman active avoidance (USAA) (Sidman, 1953a,b). While these studies show that aversive PIT is possible, the studies were mostly done in dogs and there have been no studies of the brain mechanisms involved.

We have designed a new PIT task for studies in rats. In this task, USAA (two-way shuttling) is enhanced by the presentation of an aversive Pavlovian CS. Two-way shuttling is more compatible with the repertoire of species-specific defensive responses (see Bolles, 1970) of rodent subjects than are the responses used in past studies, such as bar pressing or wheel turning (Weisman and Litner, 1969). Rats first receive excitatory PTC, followed by USAA training with the two-way shuttling response. During the test phase, following a baseline period of shuttling extinction, the Pavlovian CS is presented and the effect on shuttle rate is observed in the absence of shock.

The first study reported below examined aversive PIT using a basic approach, which was elaborated on in later studies. For instance, in order to identify the ideal baseline for test sessions, experiment 2 compared PIT when CS testing occurred following 5, 40, or 60 min of shuttling under extinction. Experiment 3 evaluated PIT under conditions where CS tests were triggered once baseline rates of shuttling reached specified criteria (e.g., 3 vs. 2 vs. 1 responses per minute or RPMs). Experiment 4 examined whether PIT magnitude is related to footshock intensity experienced during PTC (e.g., 0.35, 0.7, or 1.4 mA). Experiment 5 verified the dependence of aversive PIT on associative learning. Together these studies characterize a PIT task in rats that is aversively-motivated and distinct from conditioned suppression because the CS invigorates defensive instrumental responding, therefore, making it more comparable to appetitive PIT studies.

## General methods

### Subjects

All subjects were male Sprague–Dawley rats (Hilltop Lab Animals, Scottsdale, PA) weighing between 260 and 340 g at the start of the experiment. Subjects were housed in standard plexiglass cages with paper bedding and were exposed to a 12:12 light:dark schedule. Housing and care met with current ALAC standards and were comparable between the two locations where research was conducted in this paper: New York University (New York, NY) and the Nathan Kline Institute for Psychiatric Research (Orangeburg, NY). The only difference in housing was that subjects at Nathan Kline were housed 2 per cage while those at NYU were housed individually. Subjects had free access to food (standard chow) and water while in their home cages. The different housing conditions did not appear to impact PIT and other behavior quantified in the studies below as comparable effect sizes were obtained at both institutions.

### Apparatus

Subjects underwent PTC or other treatments (e.g., unpaired CS and US presentations) in standard training chambers (context A) manufactured by Coulbourn Instruments (model no H10-11R-TC; Whitehall PA). Stainless steel grid floors carried the current for the scrambled footshock unconditioned stimulus. The chambers were equipped with 5 ohm speakers mounted to the wall for delivery of the 5 khz tone CS used in the studies. Subjects underwent USAA training and PIT testing in context B, which consisted of shuttleboxes manufactured by Coulbourn (model no H10-11R-SC) in a different room. These chambers had 5 ohm speakers mounted on both sides and the stainless steel grid-floor delivered the footshock US. A metal panel partitioned these chambers with a threshold cut away to allow for the shuttling response, which was measured with infrared detectors on either side of the threshold. In some of experiments reported below, tests to determine freezing to the CS were added to the experimental design and conducted at the end of the study in a third set of chambers (context C) which were identical to context A, but in a different room and made to be further distinct from context A by adding (1) striped patterns to the walls, (2) a solid plastic floor over the grid, and (3) peppermint scent in the trays. All chambers had video cameras for recording the session footage for later analyses.

### Procedure

#### Pavlovian threat conditioning (PTC)

On day one of the study, subjects received PTC in context A. A 5-min baseline preceded three CS-US pairings separated by a variable inter-trial interval averaging 3 min. Each tone CS (5 kHz, 30 s) coterminated with a scrambled footshock US (0.7 mA × 1 s, unless otherwise noted). The total session duration was 15 min, after which, subjects were removed from the chambers and returned to the colony.

#### Unsignaled sidman active avoidance (USAA)

In the next phase of the study (within 72 h of PTC), subjects received 15 USAA training sessions (4–5 sessions/week) in context B. A different context from PTC was used in order to cleanly evaluate the impact of the CS on the USAA behavior. Each session was 25 min in duration. Subjects received a scrambled footshock (either 0.7 or 1 mA, 0.5 s) every 5 s unless a shuttle response was performed (i.e., a shock-shock or S-S interval of 5 s). Shuttling postponed the next shock by 30 s (i.e., a 30 s response-shock or R-S interval) and subjects were provided with feedback in the form of a 0.3 s blinking house light with each shuttling response. Shuttling during the S-S interval was considered an escape and shuttling during the R-S interval was considered an avoidance response. At the end of these sessions the house lights turned and remained off, subjects were then removed from the chamber and returned to the colony for the remainder of the day. Poor avoiders, rats failing to exhibit at least 20 avoidance responses on two consecutive days (Lazaro-Munoz et al., 2010), were removed from the study following session 10 of USAA training.

#### Pavlovian-to-instrumental (PIT) testing

Following USAA, subjects underwent PIT testing. The time course between USAA and the test phase as well as the number and spacing of test sessions was varied in the studies below. These manipulations were included (1) to develop optimal testing procedures and (2) to simulate the necessary recovery time (and potential retraining) for future studies involving brain manipulations that require surgery. The specific details of the test phase for each study will be described below.

### Statistical analyses

For consistency, all PIT results from experiment 3–5 are presented as percentage of baseline responding. Prior to experiment 3 data are presented in terms of the number of responses per minute. Percent calculations for Experiment 1 and 2 data are additionally presented for comparison purposes. Data were analyzed using IBM SPSS v21 or GraphPad Prism v5.01. Figures were created in GraphPad Prism v5.01. Analyses were done mostly using between-subjects or split-plot analysis of variance (ANOVA). Follow-up *post-hoc* tests included the Bonferroni correction to adjust for alpha inflation. *T*-tests were conducted when appropriate. In all cases an alpha level of *p* = 0.05 was applied to all statistical decisions.

## Experiment 1: initial aversive PIT protocol

No widely-accepted aversive PIT protocol exists in the literature, especially using rat subjects. However, a handful of older studies demonstrated the basic aversive PIT effect in dogs and were used to guide the development of our initial rodent assay (Rescorla and LoLordo, 1965; Rescorla, 1968; Overmier and Payne, 1971; Overmier and Brackbill, 1977; Patterson and Overmier, 1981). As in appetitive studies, three phases comprised the experiment: (1) PTC, (2) USAA training, and (3) PIT testing. We used a standard auditory PTC procedure in order to relate PIT findings to a large body of previous PTC work (Johansen et al., 2011a,b). We also chose an USAA procedure to establish a steady rate of instrumental responding (two-way shuttling), as in many of the early aversive PIT studies (e.g., Rescorla and LoLordo, 1965). Parameters for the shock US (1 mA × 0.5 s), intervals (5 s S-S and 30 s R-S) and feedback stimulus were chosen because they were effective in past studies (Bolles and Popp, 1964; Weisman and Litner, 1969; Patterson and Overmier, 1981). Procedures for testing transfer, the effect of a Pavlovian CS on instrumental response rates, varied considerably in past studies. For instance, approximately half of the published studies tested transfer with the subjects first responding in (avoidance) extinction (e.g., Rescorla and LoLordo, 1965; Overmier and Payne, 1971), whereas the other half tested transfer effects during ongoing (reinforced) avoidance training (e.g., Rescorla, 1968; Patterson and Overmier, 1981). Transfer tests that included shock may have been designed to take advantage of “warm-up” effects, where instrumental responding increases once subjects experience a shock (Overmier and Brackbill, 1977), and also to prevent rapid avoidance extinction (Weisman and Litner, 1969). Tests conducted in avoidance extinction were likely designed to prevent confounding influences of shock presentations on response rates during CS presentations. With our initial PIT protocol, we sought to balance these considerations. The PIT test session began as a standard USAA training session with shocks. However, once 15 successful avoidance responses were emitted, shockers were turned off and animals were allowed to respond in extinction. PIT was assessed by comparing response rates during three alternating CS and CS-free intervals. Each interval lasted as long as it took to complete 10 shuttles. In addition, since this protocol should be amenable to brain manipulations that could potentially require a surgery/recovery period, we examined the utility of a 5-session USAA “retraining” period after a 2-week break following avoidance training, but before PIT testing.

### Results

**Figure A1:**
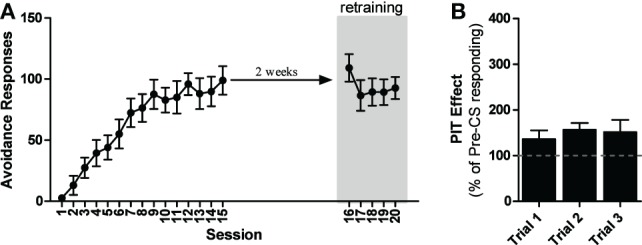
**(A)** Acquisition of shuttling during the Sidman avoidance phase and retraining (shaded gray) following a 2-week break after session 15. Data are presented as total avoidance responses per session. **(B)** Data from the test phase are presented in terms of the percentage of baseline responding for each of the 3 trials for this test session.

Rats received PTC, then USAA training followed by a 2-week homecage rest period, then USAA retraining and finally a PIT test. Poor avoiders were excluded after 10 USAA training sessions, as described previously (Lazaro-Munoz et al., 2010). Retraining appeared unnecessary; USAA performance did not drop off during the 2-week rest period. No significant difference was found between the number of avoidance responses performed during session 15 vs. session 16 (Paired *t*-test: *t*_(9)_ = 1.1, *p* = 0.30; Figure A1).

**Figure 1:**
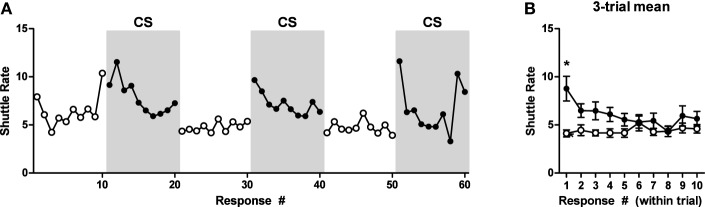
**PIT test data for experiment one. (A)** Shuttle rate for each baseline and CS (shaded gray) period. **(B)** Responding is averaged over trials. ^*^Indicates significance at the 0.05 alpha level.

Data from the test phase are presented in Figure 1 and are shown for each baseline and CS period in the left panel. An ANOVA on these data found that there was no effect of trial, therefore, the data were collapsed across this factor and are presented in this form in the right panel for clarity. Shuttling rates during the CS were generally increased relative to the baseline period. A within-subjects repeated measures 2 (Interval: Baseline or CS) × 10 (Response) ANOVA confirmed this impression, revealing a significant main effect of recording interval (Baseline or CS), ANOVA: *F*_(1, 9)_ = 11.14, *p* = 0.009. No other significant effects were obtained by this analysis. These data are also presented in terms of percentage of baseline responding in Figure A1B.

### Discussion

USAA responding after 15 training sessions is maintained during a 2-week homecage rest interval and retraining of the response is not necessary. Aversive Pavlovian CSs can facilitate USAA rates in rats. The magnitude of this facilitation was modest with the current protocol, perhaps because USAA rates were near ceiling at the outset of PIT testing because of shocks early in the PIT test session that were meant to facilitate warm-up of the USAA response. However, instrumental response extinction may help to prevent ceiling effects and improve assessment of aversive PIT. Indeed, appetitive PIT studies often use this strategy (Holland and Gallagher, 2003; Corbit and Balleine, 2005).

## Experiment 2: does the depth of avoidance extinction affect aversive PIT?

This study evaluated baseline parameters during the PIT testing phase. Because different amounts of pre-CS extinction can have direct impacts on PIT strength (Dickinson et al., 2000), it is important to determine the optimal point at which CS presentations influence shuttling. In the appetitive PIT task described above, Corbit and Balleine (2011) used a 4 min baseline of extinction prior to presenting the Pavlovian stimuli, but this may not apply to aversive PIT. In order to assess the optimal response rate for observing PIT in our task, we evaluated the impact of allowing rats to respond in extinction (no shocks) for 5, 40, or 60 min prior to presenting the CS. Rats received PTC and USAA training as above, and poor avoiders were excluded from analysis. Because Experiment 1 demonstrated that retraining was unnecessary after the 2-week training-to-testing interval, this phase was omitted from the protocol. For PIT testing, once 5, 40, or 60 min had elapsed, the CS was presented continuously until 10 shuttles were completed. Pilot experiments suggested that testing PIT once per session produced more reliable effects, thus, only one CS presentation was delivered in each PIT test session. PIT was tested on two consecutive days and shuttling rates during CS presentations were compared to rates during the 5 min preceding the CS.

### Results

**Figure 2:**
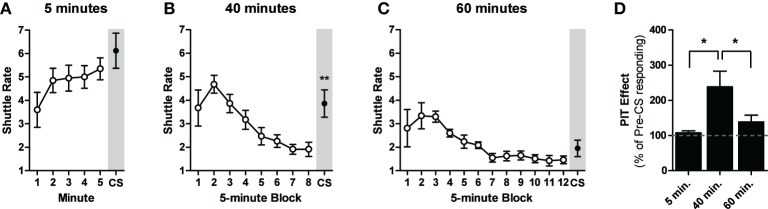
**Shuttle rates for the test phase of experiment two for subjects given either 5 (A), 40 (B), or 60 min (C) of avoidance extinction prior to the CS presentation (shaded gray). (D)** Data from the PIT tests are presented as percentage of baseline responding for all groups. ^*^Indicates significance at the 0.05 alpha level. ^**^Indicates significance at the 0.01 alpha level.

One animal from the 5-min group and two from the 60-min group were excluded from the analysis due to a failure to shuttle during PIT testing. Although response rates began lower than with our initial PIT protocol, animals in all three groups retained the USAA memory over the 2-week rest period and there were no group differences in USAA performance during the first 5-min of PIT testing [ANOVA: *F*_(2, 25)_ = 2.14, *p* = 0.14]. Significant within-session USAA extinction was observed only in the 40- and 60-min groups [ANOVAs: *F*_(7, 63)_ = 13.40, *p* < 0.01; *F*_(11, 77)_ = 6.49, *p* < 0.01]. CS presentations failed to facilitate shuttling after five [Paired *t*-test: *t*_(9)_ = 1.1, *p* = 0.28; Figure 2] or 60 [Paired *t*-test: *t*_(7)_ = 1.8, *p* = 0.11] min of USAA extinction. However, identical CS presentations after 40 min of USAA extinction significantly enhanced shuttling rates [Paired *t*-test: *t*_(9)_ = 3.9, *p* < 0.01]. Data from the test phase are also presented as percentage of baseline responding in Figure 2D.

### Discussion

These results suggest that a moderate amount of USAA extinction is optimal for detecting aversive PIT effects. The absence of PIT after 5 min of USAA extinction is likely due to a ceiling effect. No significant extinction of shuttling occurs during the 5 min and high response rates likely leave little room to observe a significant increase in responding. With our training protocol, 40 min of USAA extinction significantly reduces responding, but does not produce a cessation of shuttling. CS presentations at this point appear to reinvigorate responding to pre-extinction levels. It is unclear why CS presentations fail to facilitate shuttling after 60 min of USAA extinction. With this protocol, shuttling clearly reaches the floor well-before the CS presentation. Perhaps aversive CSs can facilitate ongoing behavior only. Lastly, these experiments demonstrate that USAA performance is retained after a 2-week training-to-testing interval and retraining is unnecessary. They also demonstrate that shocks are not needed during the PIT test to trigger USAA responding.

## Experiment 3: is there an optimal response rate for aversive PIT?

Experiment 3 examined whether testing conditions could be improved from a fixed 40-min USAA extinction baseline to a more sensitive and behavior-tailored approach. Because Experiment 2 suggested that pre-CS response rate is an important determinant of PIT strength, we designed a test in which the CS test was triggered when shuttling rates fell below specific response rates during USAA extinction. Methods were as described above except for testing procedures, which are described below. Additionally, the US intensity during the USAA phase was 0.7 mA instead of 1 mA. Experiments 3 and 4 were run at a different institution from where the other studies reported here had been conducted. An effort was made to keep general training parameters as consistent as possible. However, the different experimenters inadvertently used different shock intensities during the USAA phase.

### PIT testing

One day after session 15 of USAA training, rats received two PIT tests over 2 days. Subjects were matched for performance during the last 2 sessions (14 and 15) of the USAA phase for assignment into groups. Subjects were placed in the avoidance chambers (context B) and shuttled under extinction for 15 min. This was done because subjects sometimes require a “warm up” period—the fixed interval ensured that the start of the test session included USAA extinction. At this point (i.e., 15 min into the test), the CS presentation was rate dependent for each subject. Shuttling rates were monitored by the Coulbourn Graphic State 3 software, which triggered the CS when response rates fell below a specific threshold for two consecutive minutes. We sought to identify the ideal rate trigger for maximizing the aversive PIT effect. Since rats showing the greatest PIT effect in Experiment 2 averaged ~2 rpms, we therefore, evaluated rate triggers of 3, 2, and 1 rpms—slightly higher and slightly lower than the apparent optimal rate. Once presented, the CS remained on until the subject performed 10 responses, as previously described. PIT effects are expressed as the percentage of baseline responding. Since each subject's behavior determined the CS duration, pre-CS baselines were calculated for an equivalent period of time. For example, performing 10 responses during the CS in 60 s would result in the use of the 60 s prior to the CS presentation as the baseline period for that subject's test.

### Results

**Figure 3:**
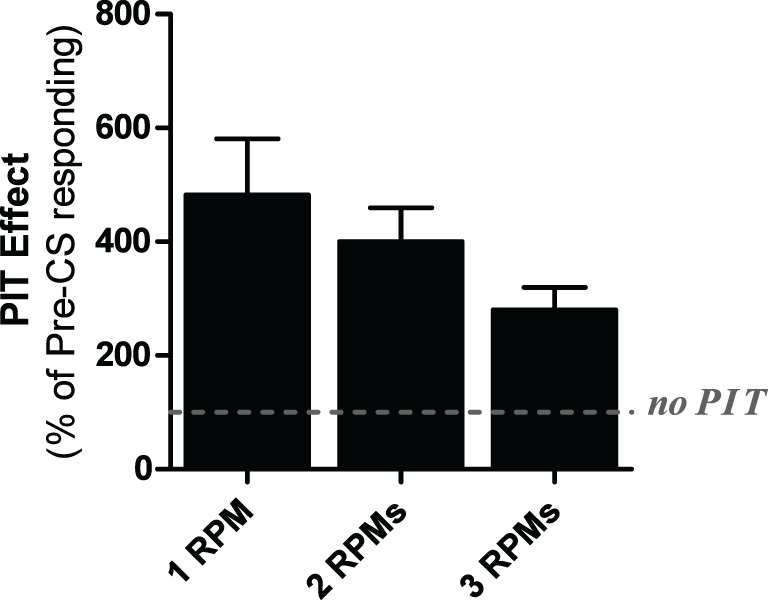
**PIT test data from experiment three**. Data are presented in terms of percentage of baseline responding for subjects presented with the CS when avoidance responding reached 3, 2, or 1 responses per minute. Baseline intervals were matched in duration to the time required to terminate the CS on each test for each subject.

Four rats were excluded from the analysis due to a lack of shuttling during the test phase. Test data are shown in Figure 3, collapsed across the two test sessions. A One-Way ANOVA found that the groups did not differ [ANOVA: *F*_(2, 33)_ = 1.41, *p* = 0.26]. However, because the assumption of homogeneity of variance was violated (Levine's test for homogeneity of variance: *F*_(2, 33)_ = 5.97, *p* < 0.05) a non-parametric Kruskal-Wallis test was conducted which produced the same outcome (Kruskal-Wallis test: *p* = 0.45). This analysis indicates that all groups performed comparably during the test phase and that the different trigger rates did not produce different transfer rates.

**Figure A2:**
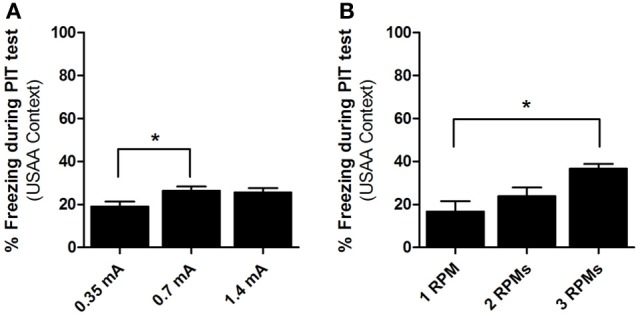
**Percent time freezing during CS presentations for the PIT testing phases in experiment 3 (A) and experiment 4 (B)**. ^*^Indicates significance at the 0.05 alpha level.

Three subjects were eliminated from video analysis of PIT testing due to recording errors. A One-Way ANOVA found that baseline freezing during the first 2 min of testing did not significantly differ *F*_(2, 34)_ = 0.812, *p* > 0.05, therefore, these data are not presented. Freezing behavior during the PIT tests can be seen in Figure A2. A separate One-Way ANOVA found that responding during the CS was significantly different across groups *F*_(2, 34)_ = 4.164, *p* = 0.024. *Post-hoc* analyses with the Bonferroni correction revealed that group 1RPM froze significantly less than group 2 rpm (*p* = 0.049). No other significant effects were found.

### Discussion

This study evaluated the impact of extinguishing pre-CS shuttling rates to 3, 2, or 1 rpms before testing the influence of the CS on behavior. While this manipulation failed to produce any effects on PIT strength directly, useful information for optimizing our PIT task can still be gleaned from these results. First, this method of testing produced stronger PIT effects than the fixed 40-min test used in Experiment 2. Because previous studies suggested that the optimal rate for PIT testing is at ~2 rpm and the current study found that driving rates any lower produced variability problems, we decided to use the 2 rpm trigger test protocol for all future studies. Moreover, while not statistically significant, the 3 rpm group showed a trend toward weaker PIT (without much difference in variability) compared to the 2 rpm group, further adding to the decision to use the 2 rpm test in future studies.

## Experiment 4: does the strength of the pavlovian memory affect aversive PIT?

To examine whether the strength of the PTC memory influences the magnitude of aversive PIT, different groups of subjects received PTC with various US intensities. On the one hand, subjects given CS-US training with very strong USs may be more motivated and show stronger PIT than subjects trained with weaker USs. On the other hand, subjects trained with stronger USs may exhibit more robust reactive defensive behavior (e.g., freezing), which could interfere with PIT. Subjects were treated as described in experiment 3 except that during PTC they received either 0.35, 0.7, or 1.4 mA USs. All subjects were tested with the 2 rpm triggered PIT test used in Experiment 3. Finally, in order to verify that the US intensity manipulation had succeeded, a separate PTC memory test was conducted in Context C one day after the completion of PIT testing. Freezing was assessed during 3 non-reinforced CS presentations (3 min. ITI); note that context C was a standard Pavlovian training chamber and that no avoidance response was available in this context.

### Results

**Figure 4:**
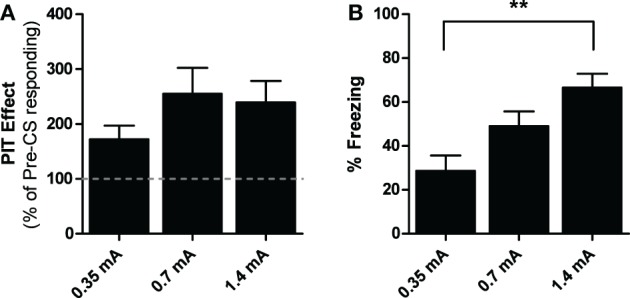
**(A)** PIT test data from experiment 4 are presented in terms of percentage of baseline responding for each group. **(B)** Freezing data from the follow up test conducted in context C 24-h after PIT testing concluded are presented for each group as mean percent time spent freezing to the 30-s CS over three trials. ^**^Indicates significance at the 0.01 alpha level.

Three subjects were eliminated from the analysis due to zero pre-CS shuttling during PIT testing. Data from the test phase are presented below in Figure 4A as percent of baseline responding. While subjects trained with the weaker US appear to show somewhat weaker PIT, a One-Way ANOVA found no differences between the groups [ANOVA: *F*_(2, 36)_ = 1.23, *p* = 0.31]. Freezing data from the follow-up test in Context C are found in Figure 4B and show mean CS-elicited freezing (percent time). One-Way ANOVA revealed a significant main effect of group [ANOVA: *F*_(2, 41)_ = 9.57, *p* < 0.01]. Bonferroni *post-hoc* tests found that while no difference was seen between groups 0.35 and 0.7 mA (*p* = 0.12), or between groups 0.7 and 1.4 (*p* = 0.09), a significant difference was found between groups 0.35 and 1.4 mA (*p* < 0.01). These results indicate that defensive behaviors such as freezing were proportional to previous US history, even following PIT tests, despite the finding of no such ordering in PIT strength itself. Thus, the magnitude of aversive PIT effects does not appear to be related to the strength of the PTC memory, at least within the behavioral ranges we tested.

Three subjects were eliminated from video analysis of PIT testing due to recording errors. A One-Way ANOVA found that baseline freezing during the first 2 min of testing did not significantly differ *F*_(2, 36)_ = 0.765, *p* > 0.05, therefore, these data are not presented. Freezing during presentation of the CS can be seen in Figure A2. A One-Way ANOVA on these data found a significant effect for group, *F*_(2, 36)_ = 6.865, *p* < 0.01. Bonferroni corrected *post-hoc* analyses showed that while the difference between freezing in groups 0.35 and 0.7 was not significant (*p* = 0.6), group 0.35 froze significantly less than group 1.4 (*p* < 0.01). The difference between groups 0.7 and 1.4 was close to significant (*p* = 0.07).

### Discussion

This study found that training with different US intensities during PTC did not have a significant influence on aversive PIT. Subjects that had the CS paired with a weak (0.35 mA), moderate (0.7 mA) or strong US (1.4 mA) during PTC all showed comparable PIT. However, these groups did demonstrate freezing commensurate with the US intensity used during PTC in the follow up test, which took place in a context with no avoidance response available. This difference between shock intensity effects on Pavlovian defensive reactions and instrumental avoidance responding agrees with previous reports showing that availability of a well-learned escape response reduces defensive reactions such as freezing and that the strength of a Pavlovian US does not factor into the vigor of avoidance behavior (McAllister and McAllister, 1991). Because PIT is not significantly influenced by US intensity, we chose to use 0.7 mA for the PTC shock value in future studies in order to keep the findings from these studies relatable to the large body of data collected by our lab.

## Experiment 5: does aversive PIT depend on pavlovian associative learning?

This final study assessed whether or not the PIT effect produced by our optimized parameters requires associative learning during the Pavlovian phase. To address this question subjects were trained and tested using the parameters suggested as ideal by the studies above. Specifically, the 30 s CS was paired with a 0.7 mA shock US. Following USAA using a 1.0 mA US, subjects were tested using the 2 rpm trigger protocol in the manner previously described. PIT effects were expressed as the percent of baseline responding. Subjects in this paired group were compared to naïve control subjects (i.e., subjects that did not receive PTC, only context exposure) as well as unpaired control subjects in order to test for behavioral selectivity (i.e., subjects that received an explicitly unpaired arrangement between the CS and US in the PTC context). Additionally, subjects were given another round of PIT tests identical to the first, but occurring 2 weeks later, in order to mimic the time that would be required to recover in an experiment involving surgical treatments.

### Results

**Figure 5:**
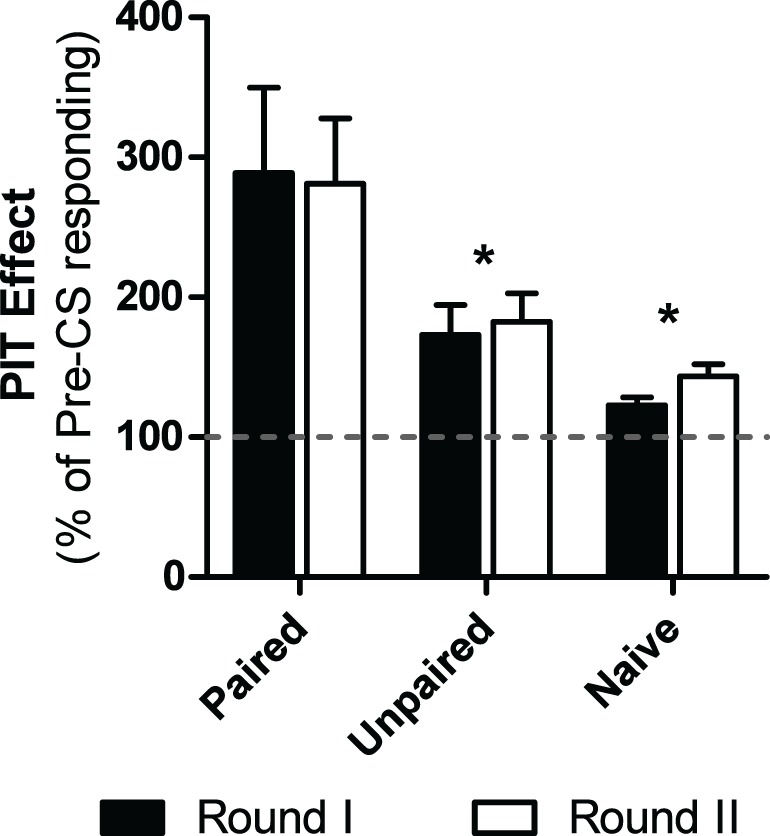
**Percentage of baseline responding during the PIT tests for each group in experiment 5**. Black bars depict PIT during the first round of PIT tests while the white bars do the same for the next round of PIT tests, which took place 2-weeks later. ^*^Indicates significance at the 0.05 alpha level.

Five subjects were excluded from analyses due to performing zero shuttles during the baseline. Of the remaining subjects, those treated with paired CS-US trials during the PTC phase showed a significant enhancement of shuttling during the CS in PIT testing compared to unpaired and naïve subjects (see Figure 5). Furthermore, this did not change over the two rounds of tests. These impressions were confirmed with a Test (Round I or Round II) × Group (Paired, Unpaired or Naïve) split plot ANOVA which revealed a significant main effect of group [ANOVA: *F*_(2, 15)_ = 6.59, *p* < 0.01]. Additionally no main effect for test [ANOVA: *F*_(1, 15)_ = 0.08, *p* = 0.78] nor an interaction between these two factors were found [ANOVA: *F*_(2, 15)_ = 0.08, *p* = 0.92]. Follow-up *post-hoc* analyses using the Bonferroni correction found that while the paired group showed more PIT than the other two groups (Paired vs. Naive *p* = 0.016, Paired vs. Unpaired, *p* = 0.048) the naïve and unpaired groups did not differ from each other (*p* > 0.05).

### Discussion

Stronger PIT was observed in subjects that received CS-US pairings in PTC compared to naïve and unpaired control subjects. This is an important result as it shows that the PIT effect developed in the preceding studies is dependent upon associative learning between the CS and US. Subjects that had learned an anticipatory relationship between the tone and shock exhibited enhanced motivation to act when later presented with the CS. The control groups showed some weak facilitation compared to pre CS shuttling rates. It is likely that while a strong PIT effect may depend on associative processes, facilitation may be due to varying amounts of general arousal. Furthermore, without any retraining, the PIT effect withstood the 2-week delay meant to simulate surgical recovery time allowing for a pre- and post-operative analysis of PIT in future studies. An additional benefit of this test approach for studies involving brain manipulations is the ability to match groups based on pre-operative PIT strength.

## General discussion

The studies presented here are a preamble to our goal of pursuing the neural mechanisms that underlie aversive PIT. In developing an aversive PIT task, we used appetitive PIT tasks as a guide. This approach was taken for two reasons. First, appetitive PIT has been more thoroughly studied (Estes, 1948; Lovibond, 1983; Rescorla, 1994; Hall et al., 2001; Holland and Gallagher, 2003; Corbit and Balleine, 2005, 2011; Shiflett and Balleine, 2010) and thus provides a clear framework for pursuing PIT task design. Second, much has been learned about the circuitry underlying appetitive PIT, which is sometimes assumed to apply to aversive states (Balleine and Killcross, 2006). This assumption has been supported by comparisons between appetitive PIT and conditioned suppression (Cardinal et al., 2002; Balleine and Killcross, 2006). While conditioned suppression also involves the effects of a Pavlovian CS on instrumental responses (Estes and Skinner, 1941; Hunt and Brady, 1951; Killcross et al., 1997), it differs considerably from aversive PIT. In conditioned suppression the Pavlovian CS is conditioned with an aversive US and the instrumental response with an appetitive US. The result is inhibition (suppression) rather than facilitation of the instrumental response. Comparisons between appetitive and aversive conditioning would be facilitated by an aversive PIT task in which the US in both phases is aversive and the CS increases the instrumental response rate.

Experiment 1 represented our first attempt to measure facilitation of aversive instrumental behavior by an aversive CS. This initial effort was informed by previous studies of avoidance enhancement by a Pavlovian CS (Rescorla and LoLordo, 1965; LoLordo, 1967; Rescorla, 1968; Weisman and Litner, 1969; Overmier and Payne, 1971; Overmier and Brackbill, 1977; Patterson and Overmier, 1981). We presented a previously trained Pavlovian CS (tone paired with shock) during performance of USAA. The results showed that, in contrast to conditioned suppression, the aversive CS enhanced ongoing shuttling behavior. Our procedure is thus congruent with appetitive PIT in the sense that the US has the same motivational significance in the Pavlovian and instrumental phases.

Experiment 1 established that aversive PIT can be readily studied, however, it was also clear that the procedure might not be optimal. The next several experiments thus represented an effort to improve PIT performance by making changes in the procedure.

Experiment 2 evaluated whether performance could be improved by manipulating the baseline response rate during the PIT test. In appetitive PIT, extinction of the instrumental response prior to testing the CS produces stronger PIT than testing shortly after the start of the session (Dickinson et al., 2000). As Holmes et al. (2010) point out, this is because the lower the baseline against which PIT is evaluated, the stronger the effect—if PIT is measured as an elevation score, reducing baseline increases the elevation. Our study found that including a moderate amount of USAA extinction prior to the PIT test produces the strongest PIT effect, with shuttling rates best at around 2 rpms prior to the CS.

In order to further determine the optimal response rate at which PIT should be evaluated, Experiment 3 tested a protocol in which the USAA behavior was required to reach the 2 rpm mark in order to trigger the CS presentation. This was found to produce the most robust and reliable PIT. Using the 2 rpm trigger protocol for testing, the final studies explored different attributes of the CS-US association and how they influence aversive PIT.

Experiment 4 found that US intensity during PTC was not a major determinant of PIT strength. This manipulation failed to result in any systematic effects on shuttling during PIT tests. While the lowest US intensity produced a trend toward weaker PIT, the other groups were essentially identical to each other. This suggests a non-linear relationship where some shock intensity (lower than 0.35 mA) will fail to support PIT while moderate and intense shocks result in similar performance. In a follow up test where no escape response was available, PTC shock levels influenced freezing rates. Previous studies have similarly found that avoidance behavior rates are not directly determined by US magnitude (reviewed in McAllister and McAllister, 1991). Experiment 5 showed that that associative CS-US learning is required for aversive PIT in our task. If the associative (Pavlovian) component of the effect were not necessary then the task would not be useful for our purposes. For example, if naïve or unpaired control subjects also showed PIT then it would be clear that the effect is not based on any associative processes and may just be due to general effects of an auditory cue. It should also be noted that while animals were housed either in pairs, or individually, we do not view this difference as crucial to the outcome of these studies. This is largely because comparable PIT effects were observed in experiments 3 and 5. Subjects were individually housed for experiment 3 and double housed for experiment 5. Because of the similar strength in PIT, housing effects on general anxiety are unlikely an important factor.

Finally, given the allowances for surgical manipulations later worked into the protocol, our task can be used to evaluate the roles of various brain regions in the aversive PIT phenomenon without altering the procedure. Comparisons of these findings with appetitive PIT and conditioned suppression findings would provide much insight into how the amygdala processes associative information and motivates instrumental actions.

Because one goal of this project is to compare PIT across motivational domains in studies involving brain manipulation, the procedural differences between the aversive task developed here and what is typically used in the appetitive literature should be addressed. Among these points is our relatively simple experimental design, compared to what is currently common in appetitive PIT studies. Designs commonly employed in appetitive PIT produce response enhancement on the basis of (a) shared motivational components for the class of USs associated with the CS and the instrumental response (e.g., foods), or general PIT and (b) unique sensory elements of the specific USs associated with a given CS and instrumental response (e.g., pellet or sucrose), or specific PIT. These studies typically involve three different combinations of CS-US stimuli (e.g., tone, noise, click CSs; pellet, sucrose, polycose USs) and two instrumental responses (e.g., bar pressing and chain pulling). This arrangement allows for within-subjects measures of both specific and general PIT. Importantly, the availability of multiple response choices seems the crucial factor for whether or not specific PIT is observed. Studies with only one available response were found to be producing general PIT (i.e., the effects were sensitive to lesions of the central amygdala) despite the presence of multiple food reinforcers in the design of the study. Our studies only used one CS-US arrangement and one instrumental response. Furthermore, we made no attempt to distinguish between general (or non-selective) PIT and sensory-specific (or selective) PIT. While certainly a future goal, studies involving multiple aversive USs and instrumental responses are not the ideal place to start. Because of the difficulty involved in presenting multiple, brief and perceptually distinct aversive outcomes and making numerous avoidance responses available to rats, different forms of PIT cannot be easily produced in our task.

This does not preclude the observation of different forms of aversive PIT using an avoidance-based task. Nadler et al. (2011) used an avoidance-based PIT task in humans where after learning to avoid simulated aversive outcomes by pressing specific response keys, subjects received Pavlovian conditioning in which visual stimuli were paired with the aversive outcomes from the previous phase. During a test, subjects showed general as well as specific transfer effects when presented with these visual stimuli. In this study, in contrast to studies of rodents, multiple aversive outcomes were available due to the conceptual nature of the task and its reinforcment, which took place over a simulation in which the subject was defending the fictitious country of Viltoma from enemy tanks, warships and warplanes—these were the three aversive outcomes as they represented threats to Viltoma. Similar to our task with rodents, performance of the instrumental response was negatively reinforced. Pressing the appropriate buttons during instrumental training prevented tank, warship and warplane attacks. In our task, performing the instrumental response (i.e., shuttling) resulted in the omission of an otherwise expected shock US. Despite this departure from the positive reinforcement seen in appetitive PIT studies response enhancement was still observed in our task and furthermore, both forms of PIT were produced by Nadler et al. (2011).

In summary, this series of experiments shows the development of an aversive PIT protocol that produces strong facilitation of ongoing instrumental avoidance responding. Our final protocol produces a 300–400% increase in response rate, some appetitive protocols produce a more moderate (~200%) increase (Hall et al., 2001). This robust effect has been shown to be very reliable—it has been replicated over multiple experiments as well as at different institutions. The effect has been shown to be dependent upon associative CS-US learning and has proven to be stable over a delay. This attribute makes the task amenable to experiments involving surgical manipulations evaluating the neural circuits involved in aversive PIT.

## Conflict of interest statement

The authors declare that the research was conducted in the absence of any commercial or financial relationships that could be construed as a potential conflict of interest.
